# Release of Nanoparticles in the Environment and Catalytic Converters Ageing

**DOI:** 10.3390/nano11123406

**Published:** 2021-12-16

**Authors:** Sofía Navarro-Espinoza, Diana Meza-Figueroa, Roberto Guzmán, Alberto Duarte-Moller, Hilda Esparza-Ponce, Francisco Paz-Moreno, Belem González-Grijalva, Osiris Álvarez-Bajo, Benedetto Schiavo, Diego Soto-Puebla, Martín Pedroza-Montero

**Affiliations:** 1 Departamento de Física, Posgrado en Nanotecnología, Universidad de Sonora, Hermosillo 83000, Sonora, Mexico; sofia.navarro@unison.mx; 2 Departamento de Geología, Universidad de Sonora, Hermosillo 83000, Sonora, Mexico; francisco.paz@unison.mx (F.P.-M.); belem.gonzalez@unison.mx (B.G.-G.); 3 Department of Chemical and Environmental Engineering, University of Arizona, Tucson, AZ 85721, USA; guzmanr@arizona.edu; 4 Facultad de Ingeniería Civil, Mecánica e Industrial, Universidad de La Salle Bajío, León 37150, Guanajuato, Mexico; jduarte@delasalle.edu.mx; 5 Investigación en Materiales Avanzados, S. C., Complejo Industrial, Chihuahua 31136, Chihuahua, Mexico; hilda.esparza@cimav.edu.mx; 6 Departamento de Investigación en Física, Universidad de Sonora, Hermosillo 83000, Sonora, Mexico; osiris.alvarez@unison.mx (O.Á.-B.); diego.soto@unison.mx (D.S.-P.); 7 Consejo Nacional de Ciencia y Tecnología CONACyT, Ciudad de México 03940, Mexico, Mexico; 8 Instituto de Geofísica, Universidad Nacional Autónoma de México, Ciudad de México 04150, Mexico, Mexico; benedetto@atmosfera.unam.mx

**Keywords:** vehicle catalyst, washcoat loss, sintering nanoparticles, non-exhaust emissions, refractory nanoparticles

## Abstract

A Three-Way Catalyst (TWC) contains a cordierite ceramic monolith coated with a layer of Al_2_O_3_, Ce_x_Zr_1−x_O_2_ and platinoids mixture. Under standard operation, the platinoid concentration decreases, exposing the remaining washcoat structure. After that particle release stage, the sintering process follows where the crystalline Ce_x_Zr_1−x_O_2_ solution is broken and begins to separate into ZrO_2_ and CeO_2_ phases. ZrO_2_ is released to the environment as micro and nanoparticles, while a small amount of CeO_2_ generates a new Al_x_Ce_1−x_O_2_ composite. The main effect of Ce capture is the growth in the size of the polycrystal structure from 86.13 ± 16.58 nm to 225.35 ± 69.51 nm. Moreover, a transformation of cordierite to mullite was identified by XRD analysis. Raman spectra showed that the oxygen vacancies (Vö) concentration decreased as Ce_x_Zr_1−x_O_2_ phases separation occurred_._ The SEM-EDS revealed the incorporation of new spurious elements and microfractures favouring the detachment of the TWC support structure. The release of ultrafine particles is a consequence of catalytic devices overusing. The emission of refractory micro to nanocrystals to the atmosphere may represent an emerging public health issue underlining the importance of implementing strict worldwide regulations on regular TWCs replacement.

## 1. Introduction

Automotive emissions constitute a significant source of air pollutants in urban environments [1]. Cars contribute to air pollution through (i) exhaust emissions from the combustion of gasoline in the engine (toxic gases); and (ii) non-exhaust emissions released from the wear of vehicle parts (particulate matter, PM) [2]. The PM is separated in PM_10_ (<10 μm), PM_2.5_ (<2.5 μm) and PM_0.1_ (<100 nm) according to its aerodynamic diameter. Currently, global emissions regulations have been implemented to reduce toxic gases, PM_10_ and PM_2.5_ from vehicles. For reducing traffic’s environmental impact, most gasoline engine vehicles incorporate a Three-Way Catalyst (TWC). This device converts automotive exhaust emissions by promoting the oxidation of carbon monoxide (CO) and unburned hydrocarbons (HC), as well as the reduction of nitrogen oxides (NO_x_) [3]. The first catalytic converters (introduced in 1975) were known as a two-way catalysts since they reduced CO and HC by oxidation reactions in the presence of Pt and Pd. Initially, NO_x_ reduction was processed by a mechanical system called exhaust gas recirculation. However, in 1980 the introduction of Rh featured the catalytic converter with the ability to reduce NO_x_ [4]. The dispersion of platinoids over the surface of a cerium-oxide substrate increased the performance of TWC. The specific selection of cerium was due to its vast oxygen storage and discharge capacity. Each free O_2_ molecule creates a space in this oxide and produces two Ce^3+^ (Ce4f) cations by distributing electrons to two Ce^4+^ cations (Ce4f^0^) [5]. But the pure CeO_2_ demonstrated poor thermostability and clustering at high temperatures losing its crucial oxygen storage and release characteristics [6]. One approach to overcoming these disadvantages was introducing ZrO_2_ and Al_2_O_3_ as thermal stabiliser agents [7]. Currently, TWC contains an active layer (washcoat) made of Ce_x_Zr_1−x_O_2_-Al_2_O_3_ and platinoids on a honeycomb cordierite structure (2MgO·2Al_2_O_3_·5SiO_2_) [8]. The synthetic cordierite (α-C) exhibits a low thermal expansion and is resistant to cracking or thermal shocks during rapid temperature transients. The melting point of α-C is 1470 °C, while the catalyst reactions occur at an operating temperature between 300 and 1000 °C [9]. The overcomes from chemical, thermal and mechanical stress conditions degrade the active catalytic washcoat [10].

Despite the environmental benefits attributed to the TWC, some studies report the release of Pt, Pd and Rh to the environment from this device [11,12]. Moreover, the vehicle’s exhaust system is considered the primary source of those pollutants in urban areas [13], including CeO_2_-ZrO_2_ micro and nanoparticles (NPs) [14]. However, neither the mechanism of particle release nor the indicators related to transformations within TWC have been addressed. Different methods have been proposed to elucidate and get experimental insights on how a TWC ages [15,16,17,18,19,20,21,22,23]. Nevertheless, these works do not test the TWC under actual conditions of use.

In this study, we physiochemically characterised two road-aged TWCs and compared them versus a new catalyst to (i) determine the chemical variability associated with the use, (ii) know the mineralogy related to thermodynamic changes, (iii) identify the wear condition and particle size at different points along the longitudinal axis of the catalyst and, (iv) analyse an exhaust pipe sample to examine the presence of materials associated with the TWC. The obtained experimental data are the basis for understanding the degradation and phase transformation processes of the washcoat and ceramic structures that impact catalytic devices’ performance.

To our best knowledge, this research would be the first work to provide insights about the processes and their effects generated on TWC with normal use conditions considering the physical wear and the subsequent environmental release of refractory particles.

## 2. Materials and Methods

We studied samples from three TWCs of sedan-type commercial automobiles. Samples were taken from A: brand-new (Nissan, Versa 2019), B: moderately used (Dodge, Atos 2014) and C: highly used TWC (Honda, Accord 2009). The mileages for B and C catalytic devices were 74,500 and 125,000 mi, respectively. The used B-TWC was circulating in Mexico City. In this city, vehicle emission regulations require the drivers to substitute the catalytic converter when the efficiency of toxic gas conversion is insufficient to comply with PROY-NOM-167-SEMARNAT-2016 guidelines [24]. On the other hand, the used C-TWC corresponds to a car circulating in Hermosillo without TWC replacement regulations.

**Figure 1:**
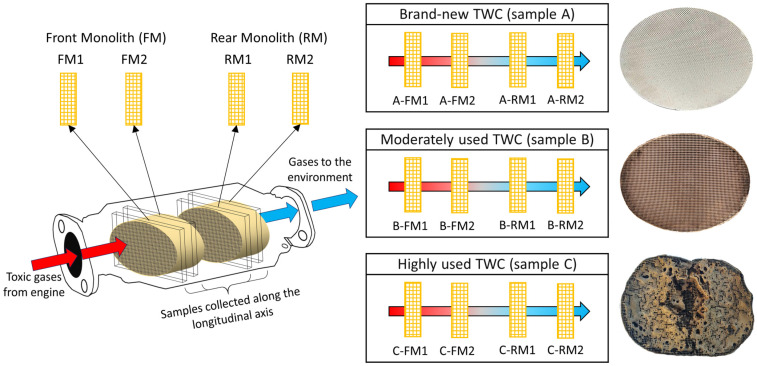
Scheme of the monolith sampling along the longitudinal axis. Labels for the four slices of TWCs were assigned from the front (FM1 and FM2) to the rear (RM1 and RM2). The gas flow direction coming from the engine is indicated with an arrow (red to blue).

TWCs features two ceramic monoliths: the front (FM) and the rear (RM) parts [3]. The FM directly receives the toxic gases from the engine, starting the gas conversions. The gases then flow to a second stage in the RM before being expelled to the atmosphere. For the physicochemical characterisation and analysis, four laminar sections were cut and labelled as FM1, FM2, RM1 and RM2, as depicted in Figure 1. From these blocks, sub-samples consisting of 0.3 × 0.3 × 0.2 mm were carefully cut perpendicular to the honeycomb structure of the ceramic monolith. A portion of the total TWC (monolith + washcoat) was pulverised in a ball mill Retsch (Bioblock) model S100. We placed ~20–25 g of sample in a high purity agate (SiO_2_) container with 250 mL capacity and 12 balls of Ø 2 cm. The powder sample was accomplished in two cycles of 6 min at 500 rpm. At the end of the second cycle, the particle size obtained was ≤45 µm. This size gives a larger surface area for exposure to acid solutions, which allows for the more efficient digestion of the sample for analysis. We had sub-samples of the washcoat after scraping the monolith surface with a stainless-steel needle. Additionally, we collected dust from the highly used TWC (most deteriorated) car exhaust pipe with a brush. In the last case, the sample amount was insufficient for performing ICP-AES and X-ray diffraction or Raman spectroscopy due to the intrinsic fluorescence. All the samples were stored in sealed bags.

### 2.1. Chemical and Mineralogical Composition

The bulk elemental concentration of the total pulverised TWCs was obtained by the certified commercial laboratory ALS Chemex Laboratories (Vancouver, BC, Canada). A powdered sample (0.100 g) was added to a lithium metaborate/lithium tetraborate flux, mixed well, and fused in a furnace at 1025 °C. The resulting melt was then dissolved in an acid mixture containing nitric, hydrochloric and hydrofluoric acids. This solution was analysed by ICP-AES (major elements) and ICP-MS (trace elements). Due to the potentially high content of Ce and Zr, we followed different digestion and measurement methods: 0.4 g of each sample was dissolved with concentrated nitric acid for half an hour to measure the Ce and Zr concentrations. After cooling, hydrochloric acid was added to produce aqua regia, and the mixture was then digested for an additional 1.5 h. The resulting solution was diluted to 100 mL with deionised water and then analysed by ICP-AES. Certified blanks and standard materials were used for quality control. Precision (%) was calculated after analysing the standard materials: AMIS0304, AMISO461, AMISO571, BCS-512 and OREAS-101b. The accuracy obtained for all samples was in the range of 92% to 106%. The LOD of major elements was 0.01% (SiO_2_, Al_2_O_3_, MgO, TiO_2_, BaO, P_2_O_5_, SrO_2_, K_2_O, CaO, Na_2_O, MnO, Fe_2_O_3_) and 0.002% for Cr_2_O_3_. The LOD of trace elements were 0.01 ppm (Cs); 0.05 ppm (U and Th); 0.1 ppm (Ce, Ga, Hf, Nb and Ta); 0.2 ppm (Rb); 1 ppm (Sn and W); 2 ppm (Zr); 5 ppm (V). The percent loss on ignition (LOI) was calculated from the difference in a sample weight (1.0 g) when the sample is placed in an oven at 1000 °C for one hour and cooled.

The mineralogical and chemical composition of TWC samples were studied with a Raman microscope (Witec, alpha300 RA, Ulm, Germany). Washcoat samples were placed on a calcium fluoride substrate (CaF_2_,13 mm Ø × 1.0 mm, Crystan Ltd., Poole, UK) with a drop of deionised water (18.2 Ω·cm, Milli-Q, Millipore, Burlington, MA, USA), and dried in a desiccator for 3 h. We used a frequency-doubled Nd: YAG laser excitation of 532 nm focused through a 50× objective. A spectral resolution of 3 cm^−1^ was achieved for this experimental setup using a 600 gr/mm grating scattered the Raman light. The measurements were taken with a 10 s accumulation time. The crystal structure of pulverised samples (monolith + washcoat) was determined using an XRD system with Bruker D8 Advance equipment. The operating conditions were 40 kV/35 mA with a Cu radiation source λ (K⍺1) = 1.5406 Å at room temperature. The range of 2θ was 10–70°, with a step size of 0.02° in a time per step of 2 s. The software Diffrac-plus EVA, supported by the International Centre for Diffraction Data (ICDD) database, was used for scan interpretation and minerals identification.

### 2.2. Physical Wear and Particle Size

We used SEM-EDS to evaluate possible physical degradation and microfractures of the TWC. In this sense, we analysed the small pieces and exhaust pipe dust with a PhenomProX desktop SEM (Thermo Fisher Scientific, MA, USA). The samples were placed on a carbon sticker in the SEM pin holder. We identified the characteristic chemical elements by EDS analysis (Quantax 200 X-ray energy dispersive spectrometer, Bruker, GmbH, Berlin, Germany). The size and morphology of the washcoat particles were analysed with a transmission electron microscope (HITACHI 7700, Tokyo, Japan) operating at 100 kV. About 50 mg of the washcoat dust were placed in 2 mL vials filled with isopropanol. The samples were then subjected to ultrasound for 10 min, and a drop was deposited on 300 mesh copper grids for the TEM analysis.

### 2.3. Data and Statistical Analysis

Statistical analysis was performed with XLSTAT 2020.5.1. (New York, NY, USA). We applied the principal component analysis (PCA) to identify the main chemical differences among the studied TWCs. This method allows the reduction of dataset dimensionality with minimal loss of information by transforming a large set of variables into a smaller one (components). As a result, the technique better interprets chemical data and samples. PCA can determine the relationship between metals and extract several independent factors with similar geochemical patterns. The suitability of the dataset was assessed by the Kaiser-Meyer-Olkin (KMO) measure and Bartlett’s test of sphericity. The KMO value was >0.62. Factors were identified by the magnitude of factor loadings: A value >0.7 implies a strong relationship, as previously reported by Vlasov et al. 2021 [25].

The raw data were processed in OriginPro 2017 (OriginLab, Northampton, MA, USA) using the Savitzky-Golay method with a 20-point window. The baseline was then subtracted with asymmetric least squares and normalised. Finally, the spectra were fitted using a gaussian deconvolution to locate the Raman and XRD peaks.

## 3. Results

### 3.1. Chemical and Mineralogical Composition

**Figure 2:**
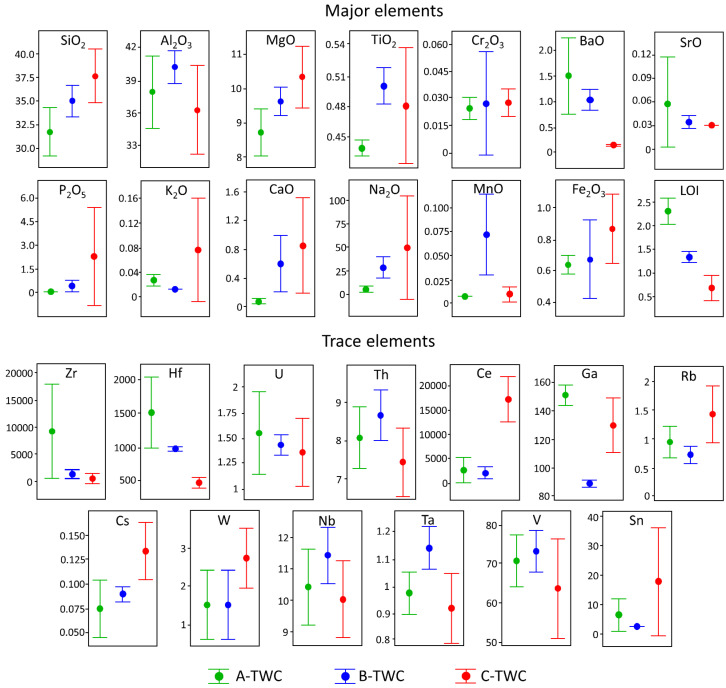
Boxplot of the major (wt%, vertical axis) and trace elements concentrations (ppm, vertical axis) of the studied samples. 95% CI for the mean, individual standard deviations were used to calculate the intervals.

The bulk composition of the monolith and refractory washcoat of the TWC is presented in Figure 2. Zr, Hf, SrO, BaO and LOI values show a decreasing trend from the new to the most used catalyst. Therefore, the diminution of Zr may be a marker for the detachment of the washcoat. Barium oxide is commonly a stabiliser to help maintain the surface area of ɣ-Al_2_O_3;_ its loss in the aged device may also contribute to the collapse of the surface area and further sintering of Ce-oxides [26]. We report the total TWC composition because the LOI is relevant data since it reflects the content of volatiles in the new and used TWC. The TWC reaches high temperatures (900–1100 °C) during regular operation, releasing the volatile components. As a result, LOI is more significant in the A-TWC than in the C-TWC, suggesting the decrease of hydrated oxides (HO). The release of volatile elements may promote mineral phase transformations in the monolith, but we recommend further studies to explore this effect.

Ce content also diminishes in the moderately used device, but in sample C-TWC, its concentration increases compared to B-TWC. This fact is related to the separation of ZrO_2_ and CeO_2_ phases. The ZrO_2_ escapes from the Ce_x_Zr_1−x_O_2_ solid solution while CeO_2_ keeps trapped in the Al_2_O_3_ layer [14,27]. The addition of ZrO_2_ improves thermal stability and inhibits the reaction of CeO_2_ with the alumina substrate [7]. Thus, the ZrO_2_ separates as nano- and microparticles [14], while the Ce is retained in the TWC, forming an Al_x_Ce_1−x_O_2_ composite (sample C-TWC) [7]. This information explains why Zr is found in higher amounts in environmental samples when compared to Ce abundance in the same samples [14]. In addition, SiO_2,_ Al_2_O_3_ and MgO (cordierite components) increase in the B-TWC sample. These compounds are concentrated after the partial release of the washcoat. However, a significant decrease of Al_2_O_3_ is then observed in the C-TWC. Al_2_O_3_ is also a central component of the washcoat, and the total Al_2_O_3_ loss impacts the bulk content, as is depicted in Figure 2.

**Figure 3:**
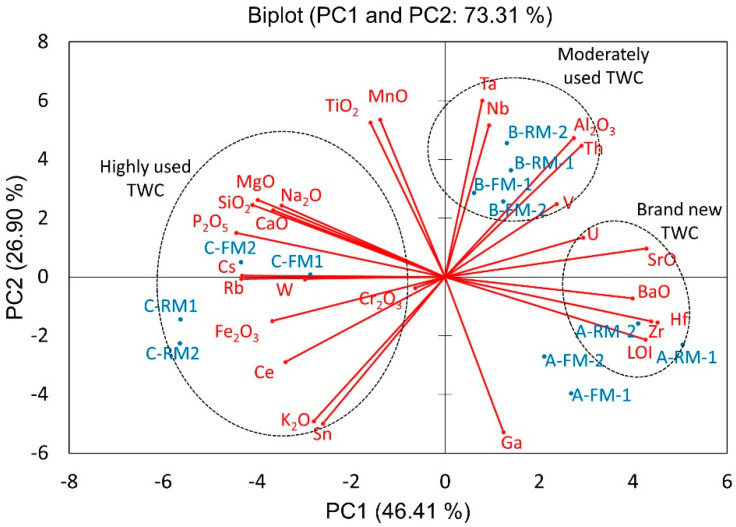
Principal component analysis (PCA) loadings of chemical composition and the studied TWCs. Enclosed ellipses correspond to chemical elements associated with each sample.

PCA splits the geochemical signatures of the three studied TWC (Figure 3). Within the obtained data set of the concentrations of metals, the first two principal components explain 73.31% of the variability. The first principal component (PC1) defined 46.41% of the total variance, dominated by Ba-Hf-Zr-Sr-LOI Ce-Cs-Rb-V-SiO_2_-Fe_2_O_3_-CaO-MgO-Na_2_O-P_2_O_5_, thus separating the brand new from the highly used catalytic converters, respectively. PC1 represents the geochemical signature of the refractory washcoat and monolith. The strongest correlative relationship of Zr, Hf and loss of ignition (LOI) in the brand new TWC reflects the unmodified refractory washcoat. The chemical ageing incorporates elements in oil additives deposited on the TWC. Typical poisons contain Ca, K, P, Mn, Pb and Zn [19]. Figure 3 shows highly used TWC with a complex geochemical signature defined by the contaminating elements. The accumulation of poisoning chemicals on the catalyst surface is one of the main reasons for its deactivation [28,29]. The C-TWC is the most deteriorated of the studied samples. The robust association with multiple trace elements indicates device poisoning and loss of the refractory washcoat after prolonged use.

The second principal component (PC2) explains 26.90% of the total variance, represented by Ga-Nb-Sn-Ta-Th-Al_2_O_3_-K_2_O-TiO_2_-MnO. The chemistry of moderately used TWC is described by PC2. The main changes in Al_2_O_3_ and Th contents are potentially attributed to phase changes in the ceramic monolith and the partial loss of the refractory washcoat. According to Christou et al. (2012) [28], the accumulation of these contaminants on the monolith results in a significant loss in its surface area (50–67%) and pore volume (40–69%). Additionally, the chemical change leads to new solid phases in the catalyst, causing the structure to collapse and clog the pores [28]. The contribution of the third principal component (PC3) is 8.4%, and it is dominated by U-W, possibly related to brand differences. The fourth principal component (PC4) is attributed to Cr_2_O_3_ with 6.87% of cumulative variance.

**Figure 4:**
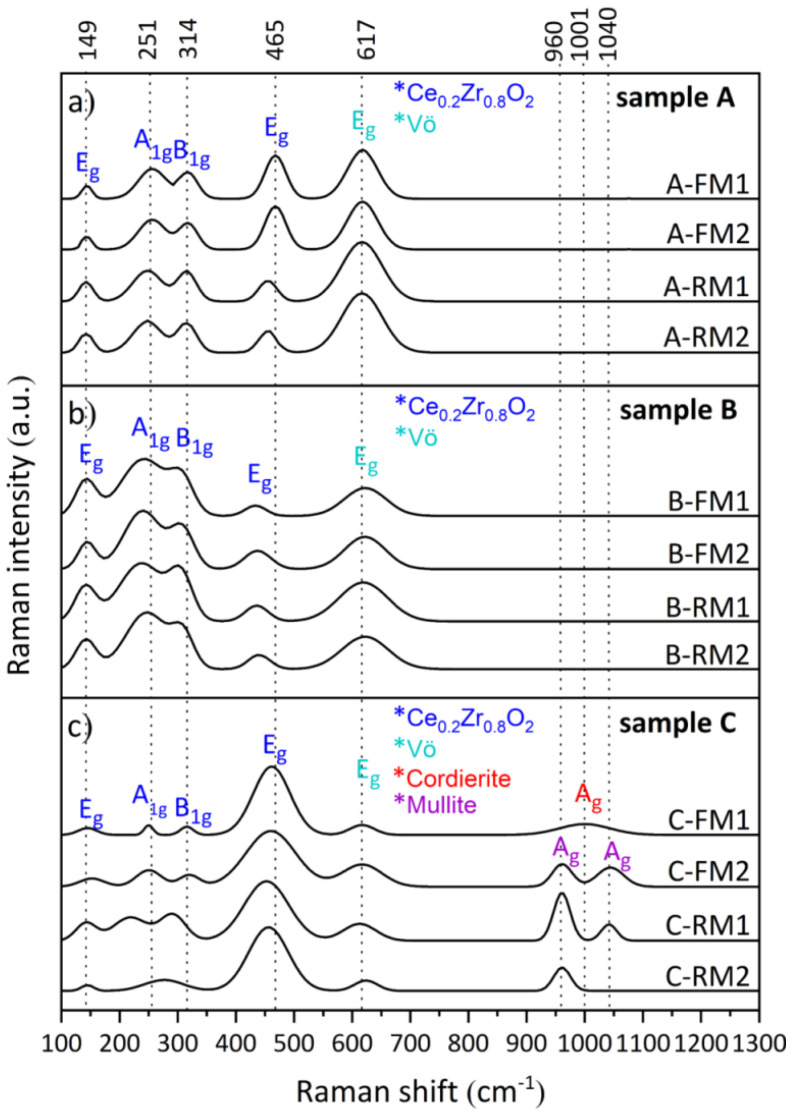
Identification of vibrational Raman modes associated with mineral phases in (**a**) new; used (**b**) B−TWC and (**c**) C−TWC. E_g_ modes at 149, 456 and 617 cm^−1^ correspond to oxygen stretching of Zr−O, Ce−O and Vö, respectively.

The Raman spectra of the washcoat exhibited a characteristic Ce_0.2_Zr_0.8_O_2_ spectrum (Figure 4a). The peak at 465 cm^−^^1^ is attributed to the symmetric stretching mode of the vibratory unit Ce-O [30]. Peaks at 145, 255 and 314 cm^−1^ are assigned to the E_g_, A_1g_ and B_1g_ vibrational modes resulting from the division of F_2g_ (from cubic CeO_2_) [31]. The peak splitting at 465 cm^−1^ is due to the distortion of cubic CeO_2_ crystals to a tetragonal structure by doping with high concentrations of Zr (>20 mol% Zr) [31,32]. Notably, the distortion of this crystal lattice produces oxygen vacancies (Vö) that are of vital importance for the catalysis process (peak at 617 cm^−1^) [30]. Therefore, the intensity of this peak (IPK) is another marker for evaluating the catalytic properties of the material since the loss of these vacancies significantly reduces its efficiency [33]. In this work, the A-TWC has the highest amount of Vö, considering that the IPK is proportional to the concentration. In this sense, this marker decreased in the B-TWC and C-TWC, indicating a reduction in the number of Vö (Figure 4b,c). Particularly, sample C-FM1 shows a peak corresponding to the cordierite support at 1001 cm^−1^ and ascribed to the vibrations of the tetrahedral sites T_2_1-T_2_3 interconnected by oxygen [34]. We detected cordierite peaks in a subsample taken from the surface because of the removal of the washcoat and the consequent exposure of the internal monolith. C-FM2, C-RM1 and C-RM2 subsamples have peaks at 960 and 1040 cm^−1^, possibly associated with Si-O stretching of the mullite structure (3Al_2_O_3_·2SiO_2_) [35,36,37]. The transformation of cordierite into mullite has been previously described in high-temperature systems. Zhang et al. (2019) studied the thermodynamic reaction in the cordierite-zirconia system when exposed to temperatures from 1150 to 1460 °C. They determined that for temperatures around 1460 °C the cordierite transforms into mullite and the glass phase of mullite and zirconia [38]. However, in our samples, the temperature reached in the used catalysts was around 1000 °C [17]. This phase transformation could be favoured by HO that lowers the melting point of cordierite. Volatile compounds and poisoning elements could generate partial fusion and the mineral phase transformation in heavily used TWC. Remarkably, these subsamples (C-FM2 and C-RM1) were melted and even distorted the honeycomb cells. This change implies the beginning of a partial phase transformation process in these sections of the ceramic monolith, as has been observed by other authors under laboratory-controlled conditions. Mullite is the only stable phase in the alumina-silica system at atmospheric pressure [39]; its structure consists of distorted chains with Al-O octahedra at the corners and centre of each unit cell. Al-O and Si-O corner-sharing tetrahedra cross-link the chains [39].

**Figure 5:**
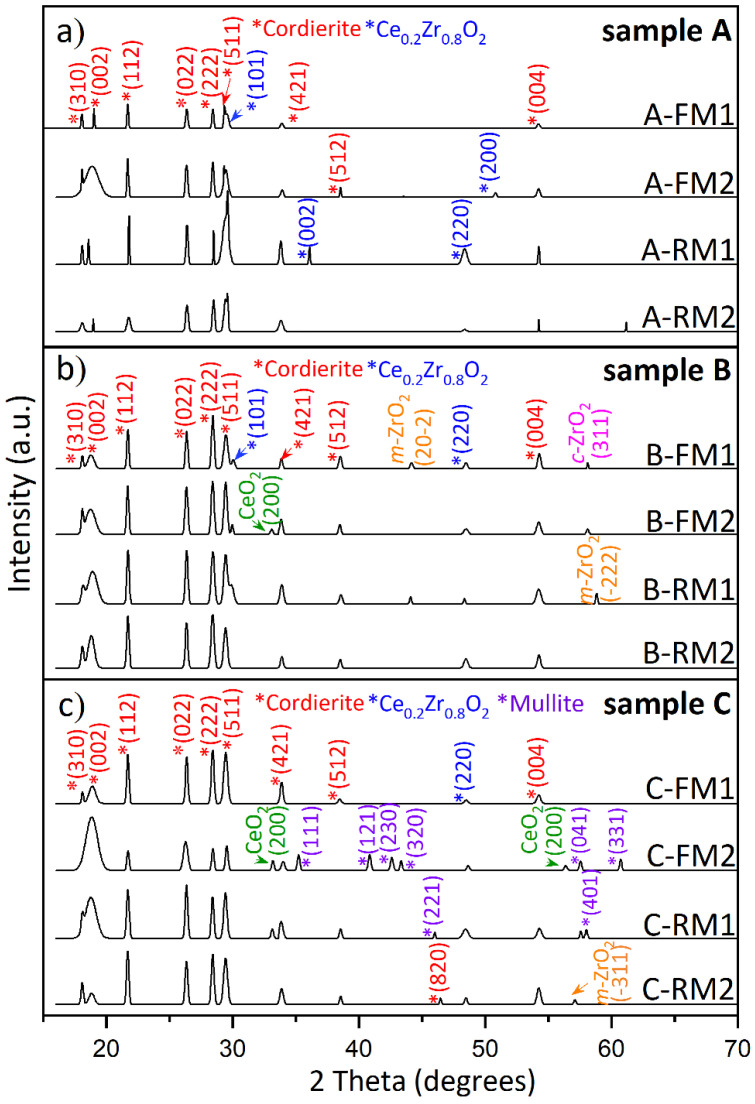
XRD diffractogram for (**a**) new; used (**b**) B-TWC and (**c**) C-TWC. Cordierite (red labels) is present in all ceramic monoliths, and phase change to mullite (purple labels) is only observed in the highly used catalyst.

The X-ray diffraction profiles (XRD) of the three TWCs are presented in Figure 5. The diffraction pattern for the A-TWC shows characteristic cordierite peaks at 18.0°, 18.97°, 21.66°, 21.72°, 26.44°, 28.46°, 29.42°, 33.76° and 38.50°, corresponding to the planes (310), (002), (112), (022), (222), (511), (421), (512), (004) (Figure 5a). The peaks of Ce_0.2_Zr_0.8_O_2,_ associated with the planes (101), (200) and (002), (220), were observed in the new TWC. In the same way, the cordierite and Ce_0.2_Zr_0.8_O_2_ peaks are observed in both the B-TWC and C-TWC. Besides, the used TWC show peaks of cubic ZrO_2_ (58.06°), monoclinic ZrO_2_ (44.13°, 57.1° and 58.79°) and CeO_2_ (33.08° and 56.34°) (Figure 5b,c), which supports the separation of ZrO_2_ and CeO_2_ phases [27].

The crystallinity of samples A-TWC, B-TWC and C-TWC is 51.52%, 41.49% and 32.53%, respectively. This decrease is dominated by the transformation from cordierite to mullite [38]. In particular, subsamples C-FM2 and C-RM1 show mineral mullite peaks at 33.34°, 35.21°, 40.84°, 42.72°, 43.09°, 48.41°, 57.59°, 58.54°, 60.81°, in agreement with the mineralogical composition observed by Raman spectroscopy in Figure 4.

### 3.2. Wear Degradation and Particle Size

**Figure 6:**
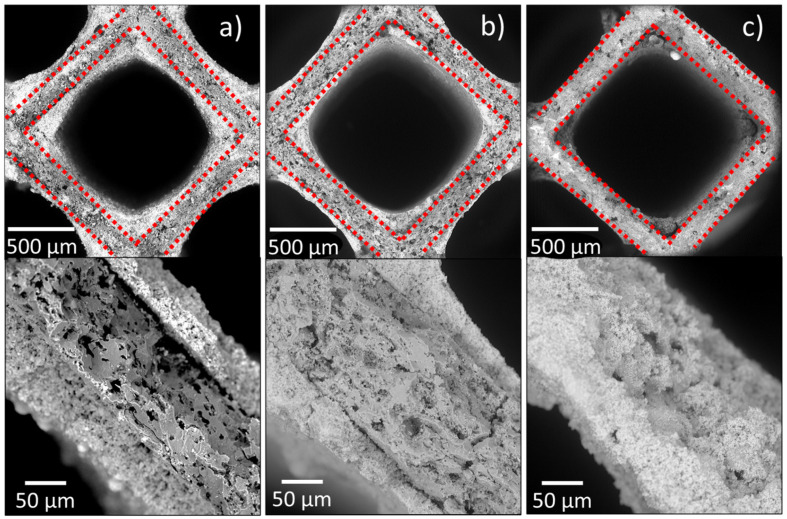
SEM images at different magnifications from (**a**) brand-new TWC, (**b**) moderately used TWC, (**c**) highly used TWC. Red dashed line separates the internal monolith and the external catalytic layer. The loss of the outer catalytic layer is proportional to the use.

SEM images show the wear degradation of the used TWCs. Figure 6a shows a cross-sectional monolith-cell of the A-TWC, where the internal cordierite structure and the washcoat can be distinguished. B-TWC displays a lightly worn catalytic layer in the cells’ central areas (Figure 6b). Washcoat loss is evident in C-TWC, and residuals of this layer are visible only at the cells’ intersections (Figure 6c). The lack of the washcoat represents two critical problems. The first is that the conversion of toxic gases does not occur, and the second is that all this material could be released into the environment. Previous works have reported the presence of platinum group elements related to traffic sources in soil/road dust [40,41,42,43], atmospheric dust [44,45,46], and the exhaust pipe [47,48,49]. More recently, the incorporation of ZrO_2_ and CeO_2_ compounds from the catalyst in environmental matrices [14] has been studied. The fracturing of refractory material may amplify the degradation and particle release [50]. SEM studies of used TWCs revealed that the ceramic support presented microfractures. Fractures were observed in B-TWC with displacements of 5.19 ± 2.17 µm, while they were not present in the A-TWC. According to Wu and Zhang, microfractures start at pre-existing defects within the monolith and then propagate to form visible macrocracks. The surfaces of the microcracks and large macrocracks are interconnected, resulting in the active material’s peel off [50]. In C-TWC, we observed irregular breakdown and loss of the surface.

**Figure 7:**
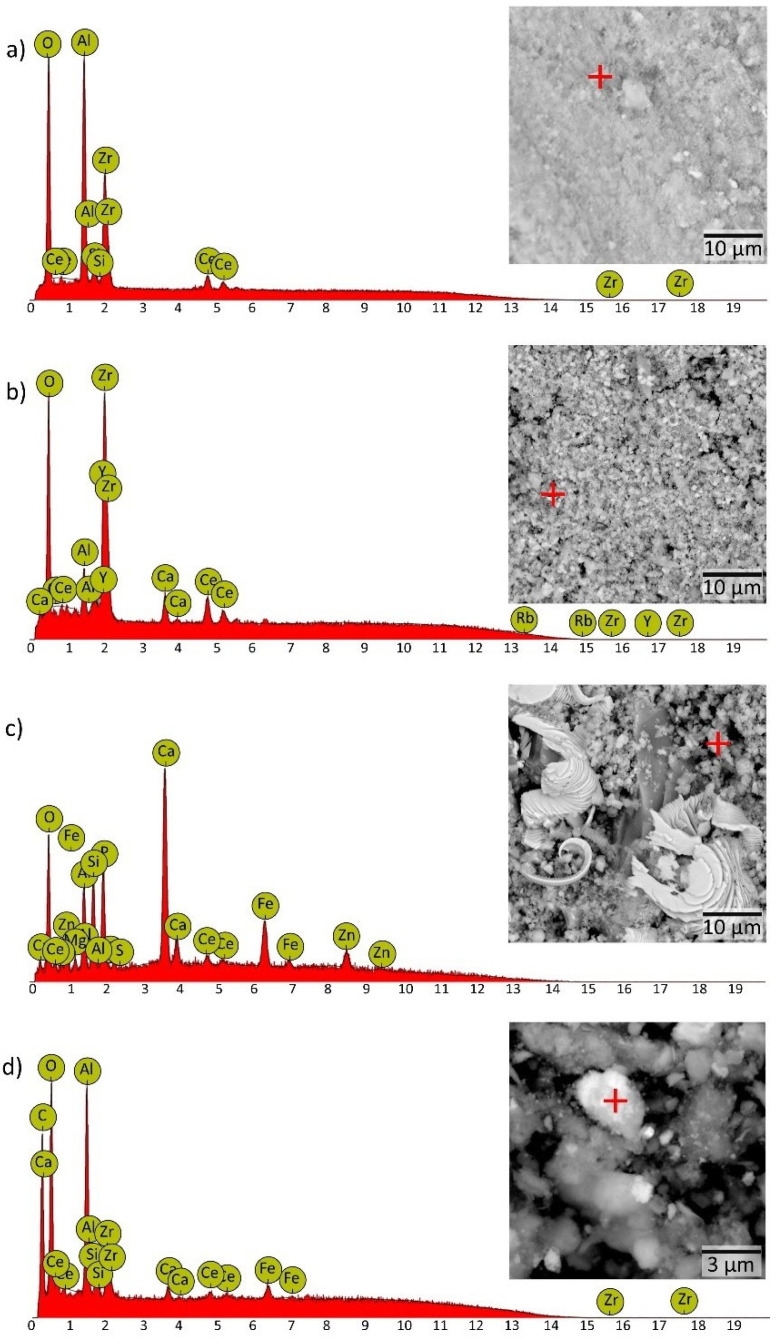
SEM image and EDS analysis for element identification from (**a**) brand-new TWC, (**b**) moderately used TWC, (**c**) highly used TWC and (**d**) pipe exhaust sample.

The EDS analysis of the external catalytic layer shows that sample A-TWC contains microcrystals made up of O, Zr, Al, Ce and Si (Figure 7a). Particles of the B-TWC are composed of Zr, O, Ce, Y, Al, Ca and Rb (Figure 7b), while C-TWC gives O, Ca, Zn, Fe, P, Al, Si and Ce (Figure 7c). These results support the chemical poisoning observed in the chemical analysis. Moreover, significant heterogeneity in the size of the C-TWC particles is evident, including fragments up to 10 µm.

**Figure 8:**
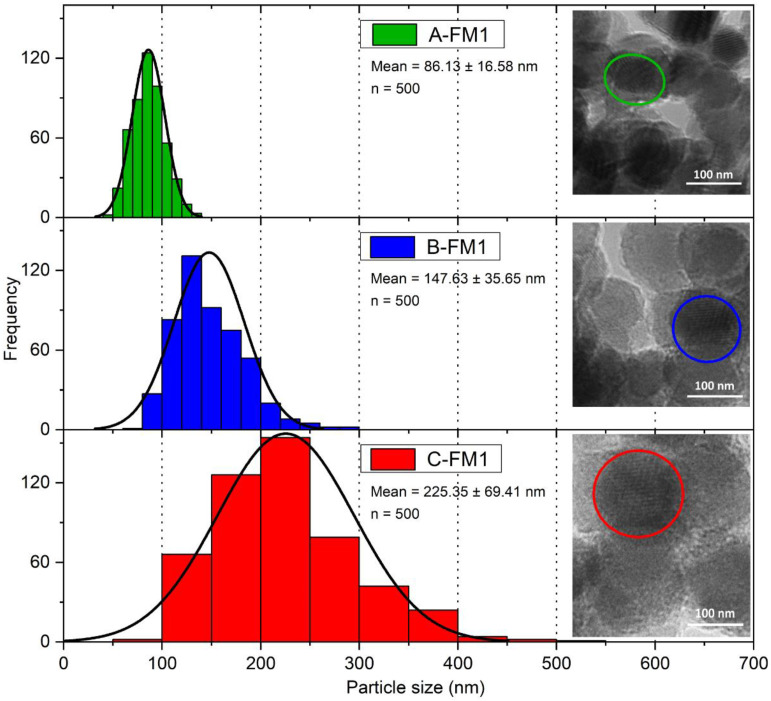
TEM images and size distribution of particles analysis from brand-new TWC, moderately used TWC, and highly used TWC. The enclosed areas correspond to the average measured particle diameter. Variation in size increases as particle grows.

Particle size measurements made by TEM are in Figure 8. Sample A-TWC, which was not exposed to high temperatures, presents an average 86.13 ± 16.58 nm diameter. In comparison, the particles of the catalysts B-TWC and C-TWC show average sizes of 147.63 ± 35.65 nm and 225.35 ± 69.51 nm, respectively. The catalytic properties of ceria compounds depend on several factors, including particle size. Reducing the particle size of a catalyst results in increased surface area and a change in its morphology, thus generating more reactive edge sites [6]. Chiefly, particles below 100 nm become nanophasic. In this manner, the density of defects increases since almost 50% of the atoms are located in the core of defects, generating many active sites for gas-solid catalysis. Moreover, diffusivity across the boundaries of the nanometer-sized interface promotes rapid kinetics of catalyst activation and reactions [6]. In our study, the used TWC presents a diameter size higher than 100 nm caused by sintering. Here, two mechanisms for the sintering of NPs may occur in the TWC: Ostwald ripening (OR) and Particle migration-coalescence (PMC) [51]. The first one involves the migration of mobile molecular species or atoms, driven by differences in free energy and local concentrations of atoms at the surface. The second one consists of the mobility of particles on the support surface followed by coalescence that leads to the growth of NPs [51]. In this work, first, we observed crystal growth mainly driven by OR in used TWC from a brand new TWC. Then, the enlarged size (possibly associated with PMC) of sintered NPs took place, reducing the adhesion and promoting the washcoat detachment from the cordierite monolith [16]. In this manner, the loss of the catalytic layer (composed mainly of Zr_0.8_Ce_0.2_O_2_) in a highly used catalyst is closely related to the sintering process.

Lastly, we collected and analysed an exhaust pipe sample to assess the release material’s contribution from TWC. The results by SEM-EDS show some agglomerates and particles smaller than 1 µm in rounded size and a bright white, indicating that it is composed of elements with a higher atomic number. EDS analysis shows that these particles contain Al, Zr, Ce and Si (Figure 7d). All these elements are representative of TWC. The presence of catalyst components in the exhaust pipe sample indicates that these refractory compounds travel through this medium propelled by gases ending in the environment. Remarkably, our group has already reported ZrO_2_-CeO_2_ compounds in complex environmental samples as micro and NPs [14]. Besides, NPs size distribution showed a predominant size range from 2 nm to 650 nm in particulate matter with variable composition from a high traffic area [52]. Inorganic agents of a refractory nature can cause respiratory diseases due to their accumulation in the lungs [53,54]. It has been reported that exposure to traffic pollution (including NPs) is related to neurodevelopmental and neurodegenerative diseases [55]. Regarding plants, NPs in dust can result in stomata clogging. Particles smaller than the diameter of stomata apertures (10–50 µm) directly enter the sub-stomatal cavity and reach the spongy parenchyma of the leaf tissue. This mechanism leads to a decrease in biomass in urban environments [56]. The above suggests that NPs coming from TWC may have significant health and ecological repercussions.

### 3.3. Limitations of the Study

The experimental results reported herein should be considered in light of the following limitations. First, the studied TWC are from different brands and have minor differences in metallic enclosures and exhaust pipes. However, all TWC have equivalent structure and functioning with two ceramic monoliths connected to positive crankcase ventilation system in 4-cylinder sedan automobiles with fuel injection system. Second, the gasoline contained various additives to boost the performance and clean the engine cycle. However, they have different formulations, mainly including P, K, Ca, Na and traces of Mn. Third, due to deterioration and loss of material in the used TWC, the amount of sample was not enough to perform a chemical analysis of platinoids. The number of samples was not enough for a complete description of PCA. Finally, just one study of each subdivision of TWC was conducted because of budget restrictions, reaching four points for the entire piece. Nevertheless, we considered it good enough for acquiring at least a qualitative experimental insight into the sintering process with ageing.

## 4. Conclusions

This article investigated three similar TWCs (one new and two used) with different degrees of wear. Physicochemical analysis showed chemical poisoning, micro-cracks and sintering of washcoat NPs. These processes are responsible for the wear and detachment of the catalytic washcoat. At an advanced stage of damage (highly used TWC), degradation of the internal monolith and chemical transformation of cordierite to mullite occur. Under these conditions, TWCs become a potential source of non-exhaust micro and NPs released into the atmosphere. Because they are in the respirable fraction (PM_10_ < 10 µm), the size of these refractory materials implies a health risk. In short, TWCs represent a crucial factor in environmental pollution after being continuously subjected to chemical, mechanical and thermal stress. The subsequent mechanism of NPs’ release generated by chemical reactions in the washcoat is not fully understood; however, the provided experimental insights will complement this picture. Future improvements to TWC components may be beneficiated from this knowledge. Besides, current atmospheric particulate matter regulations do not address PM_1_. This study shows that the emission of such particles is related to catalyst deterioration which in turn depends on local regulatory policy.
